# Editorial: Tennis: bridging tradition and progress—an in-depth analysis of the sport's evolution and its prospect for the future

**DOI:** 10.3389/fspor.2025.1620320

**Published:** 2025-05-19

**Authors:** Alexis Herbaut, Ales Filipcic, Wan Xiu Goh

**Affiliations:** ^1^ SportsLab, Decathlon, Lille, France; ^2^ Faculty of Sports, University of Ljubljana, Ljubljana, Slovenia; ^3^ Sport Science & Sport Medicine, Singapore Sport Institute, Singapore, Singapore

**Keywords:** tennis, performance, physiology, biomechanics, well-being

**Editorial on the Research Topic**
Tennis: bridging tradition and progress—an in-depth analysis of the sport’s evolution and its prospect for the future

Tennis graces the sports world with its rich blend of tradition and innovation, tracing back centuries yet persistently evolving to meet the demands of the modern era. Sporting stark transformations in terms of equipment, methodology, training, injury prevention, and data analysis, tennis has seen a consistent upswing in performance levels across the globe.

This special issue features seven articles (three reviews, three original researches, one brief report and one perspective article) aiming to dissect the factors influencing this constant evolution, weaving a comprehensive narrative of tennis's past, present, and potential future.

Competition in tennis gets tougher every year and detecting talent at an early age is crucial. Munivrana et al. compared different generic and tennis-specific agility tests on the field to determine the most appropriate to identify talented players in youth tennis categories. This study provides insightful and pragmatic recommendations for tennis coaches.

Crespo et al. conducted a systematic review of articles on the tactical and technical aspects of competitive tennis. The development of tactical-technical skills is influenced by the methods, conditions, level of athletes and areas of development. The use and creation of skill models in a complex sport such as tennis is sufficiently reliable and valid and of practical value to tennis coaches.

In tennis, the serve is considered the most essential stroke, allowing the player to take the advantage over the opponent. Jacquier-Bret and Gorce started by conducting a systematic review and meta-analysis on the kinematic key points of interest during the tennis serve. This study identified trunk inclination, shoulder elevation and lateral rotation, elbow flexion and knee flexion as the most studied kinematic parameters and proposed a mean value for each of them. Gorce and Jacquier-Bret enriched this work with another study on tennis-ranked players highlighting eleven kinematic and kinetic parameters correlated with racket velocity. These results should be valuable information for coaches to improve players' serve performance.

Very young and talented tennis players, including teenagers, compete for up to 30 weeks a year to improve their performance, while only about 20 weeks are dedicated to training and recovery. The result of this heavy workload is a significant increase in injuries among top tennis players. In a perspective article Michel et al. address this problem and offer novel approaches that can help tennis players effectively manage their well-being. They suggest several measures, such as planning training and competition schedules with adequate recovery periods, identifying and monitoring athletes' workload, using innovative training methods and proper nutrition. It is crucial for tennis players and their coaches to take a holistic approach to decision-making regarding training and competition, taking into account both health and performance-related aspects. Furthermore, studies show that one third of the tennis tournaments organized by the International Tennis Federation are played in very hot and dry or humid conditions. It is expected that even more sporting events will take place in extreme conditions in the future due to the effects of global warming. Robin et al. investigated how male tennis players competing at different quality levels cope with extreme weather conditions and what strategies they use before, during and after cooling down to maintain their health, limit performance decline and promote recovery.

As a conclusion, tennis-related research is very healthy, as shown by this special theme issue In Frontiers in Sports and Active Living and by Sampaio et al. bibliometric analysis of articles using artificial intelligence in tennis. The analysis covered various aspects of tennis, including performance, health, match results, physiological data, tennis spending and prize money. Most of the articles were published by authors from China, the United States and Australia. The predictive model suggests that the number of articles and citations on this topic will continue to increase over the next ten years.

